# Statistical analysis of publicly funded cluster randomised controlled trials: a review of the National Institute for Health Research Journals Library

**DOI:** 10.1186/s13063-022-06025-1

**Published:** 2022-02-04

**Authors:** Bright C. Offorha, Stephen J. Walters, Richard M. Jacques

**Affiliations:** School of Health and Related Research, The University of Sheffield, Sheffield, UK

**Keywords:** Intracluster correlation coefficient, Cluster randomised controlled trials, Clustering, CONSORT, Statistical methods, Recruitment

## Abstract

**Background:**

In cluster randomised controlled trials (cRCTs), groups of individuals (rather than individuals) are randomised to minimise the risk of contamination and/or efficiently use limited resources or solve logistic and administrative problems. A major concern in the primary analysis of cRCT is the use of appropriate statistical methods to account for correlation among outcomes from a particular group/cluster. This review aimed to investigate the statistical methods used in practice for analysing the primary outcomes in publicly funded cluster randomised controlled trials, adherence to the CONSORT (Consolidated Standards of Reporting Trials) reporting guidelines for cRCTs and the recruitment abilities of the cluster trials design.

**Methods:**

We manually searched the United Kingdom’s National Institute for Health Research (NIHR) online Journals Library, from 1 January 1997 to 15 July 2021 chronologically for reports of cRCTs. Information on the statistical methods used in the primary analyses was extracted. One reviewer conducted the search and extraction while the two other independent reviewers supervised and validated 25% of the total trials reviewed.

**Results:**

A total of 1942 reports, published online in the NIHR Journals Library were screened for eligibility, 118 reports of cRCTs met the initial inclusion criteria, of these 79 reports containing the results of 86 trials with 100 primary outcomes analysed were finally included. Two primary outcomes were analysed at the cluster-level using a generalized linear model. At the individual-level, the generalized linear mixed model was the most used statistical method (80%, 80/100), followed by regression with robust standard errors (7%) then generalized estimating equations (6%). Ninety-five percent (95/100) of the primary outcomes in the trials were analysed with appropriate statistical methods that accounted for clustering while 5% were not. The mean observed intracluster correlation coefficient (ICC) was 0.06 (SD, 0.12; range, − 0.02 to 0.63), and the median value was 0.02 (IQR, 0.001–0.060), although 42% of the observed ICCs for the analysed primary outcomes were not reported.

**Conclusions:**

In practice, most of the publicly funded cluster trials adjusted for clustering using appropriate statistical method(s), with most of the primary analyses done at the individual level using generalized linear mixed models. However, the inadequate analysis and poor reporting of cluster trials published in the UK is still happening in recent times, despite the availability of the CONSORT reporting guidelines for cluster trials published over a decade ago.

**Supplementary Information:**

The online version contains supplementary material available at 10.1186/s13063-022-06025-1.

## Background

Randomised controlled trials (RCTs) are the gold standard in medical and public health research when assessing the safety, clinical and cost-effectiveness of new drugs, new health technologies and new social interventions [1]. Conventionally, in RCTs, individuals are randomised to the experimental arms using either a randomisation or minimisation technique to ensure random allocation and balance in participants characteristics across the experimental arms.

Individually randomised controlled trials (iRCTs) are common, but in practice, this trial design may suffer from the potential contamination of outcomes from participants in the trial. Contamination could occur when participants in proximity are randomised to different experimental arms, there are chances that they will share their experiences of the trial which in turn may influence their outcomes. The cluster randomised controlled trial (cRCT) design can be used to minimise the risks posed by contamination [2, 3].Other rationales for using a cRCT design are maximisation of limited resources, problems with logistics, and administrative convenience [2].

A cRCT is potentially a more powerful design in handling the above-named issues, with groups of individuals (rather than individuals) randomly allocated to the experimental arms, resulting in outcome data that is clustered. Clustered data can also arise from repeated measurements over time on the same individuals in a longitudinal study. Going forward, for simplicity we have interchangeably used “cluster trials” to mean cRCTs.

In cluster trials, outcomes from a cluster/group tend to be more similar than outcomes from any other randomly selected cluster/group. This similarity (or correlation) of outcomes within a cluster is also known as the intracluster correlation. This correlation or non-independence of outcomes violates the assumptions of standard statistical methods used for assessing the effectiveness of an intervention to control, such as *t-test, F-test, chi-square test* or statistical regression methods used when researchers are also interested in adjusting for the effects of covariates and confounders, such as *linear regression, Poisson regression* and *logistic regression*. Standard statistical methods assume that the outcomes from participants in a trial are independent, most of the time this assumption does not hold in cluster trials. Ignoring the dependence among outcomes in the same cluster may lead to reduced standard errors which means—an increased value of the test statistic, smaller *P*-values and narrower confidence intervals which could increase the risk of false-positive results [1, 3, 4].Campbell and Walters [1] grouped the recognised statistical methods for analysing cluster trials into four broad approaches: (1) cluster-level analysis—using aggregate summary measures for each cluster, (2) individual-level analysis—using regression models with robust standard errors, (3) individual-level analysis—using generalized linear mixed models (random effects models), and (4) individual-level analysis—using a generalized linear model with generalized estimating equations (GEE) to estimate the model coefficients. These broad groupings relate to the way the statistical methods account for correlation among outcomes from the same cluster. The primary objective of this review is to investigate the use of these statistical methods in practice, with a focus on their prevalence.

The Consolidated Standards of Reporting Trials (CONSORT) statement was first published in 1996 to guide the reporting of iRCTs [5]. The extension of the CONSORT statement to cover cluster trials was first suggested in 2001 [6] and was then extended in 2004 [7], based on the revision of the CONSORT statement in 2001. There were still inadequacies in the reporting of iRCTs; hence, in 2010, the previous version of 2001 was updated [9]. The 2012 extension to cover cluster trials was based on this updated CONSORT 2010 statement [8]. These guidelines are meant to aid researchers in the planning, conducting, analysing and reporting of cluster trials to reduce the problems occurring from the poor reporting of cRCTs. Most of the information extracted from each trial reviewed in this study is based on this CONSORT statements extended for cluster trials.

Adherence to the CONSORT reporting guidelines for cluster trials and its impact on the quality of reporting cluster trials has attracted the interest of researchers since it was published [10–12]. The adherence to different aspects of the CONSORT statement for cluster trials is usually of interest to researchers, for example a review found that though some aspect of treatment compliance by the participants in the studies are reported, but in general, comprehensive reporting of treatment compliance by participants is poor and inadequate [12]. Another review concluded that despite the availability of the CONSORT reporting guidelines for cluster trials, the reporting of all aspects of sample size calculation was inadequate [11]. Ivers et al. [10] went a step further and investigated adherence to all the new items included in the CONSORT extension for cluster trials; they found that improvement was only evident in few aspects, while in general, the adherence to the CONSORT statement extension for cluster trials was inadequate. The success of any guideline can be measured by the rate of its implementation in practice [13].

One of the justifications for conducting this study was to contribute to the debate in the literature on the adherence to the CONSORT reporting guidelines extension for cluster trials; our focus is on the aspect of the reporting quality of the intracluster correlation coefficient in the cluster trials reviewed. It is justifiable to investigate how well the extended CONSORT reporting guidelines for cluster trials is been implemented in practice, with the aim of recommending how to improve the quality of reporting cluster trials (if necessary).

Established in 2006, the National Institute for Health Research (NIHR) is now the largest funder of public health and social research in England. The NIHR publishes its commissioned research in the online open access NIHR Journals Library which consists of five journals: *Public Health Research* (PHR; https://www.journalslibrary.nihr.ac.uk/phr/#/), *Health Services and Delivery Research* (HSDR; https://www.journalslibrary.nihr.ac.uk/hsdr/#/), *Efficacy and Mechanism Evaluation* (EME; https://www.journalslibrary.nihr.ac.uk/eme/#/), *Programme Grants for Applied Research* (PGfAR; https://www.journalslibrary.nihr.ac.uk/pgfar/#/) and *Health Technology Assessment* (HTA; https://www.journalslibrary.nihr.ac.uk/hta/#/). In 2019/2020, the NIHR awarded over £250 million to fund 310 research projects. The NIHR Health Technology Assessment (HTA) programme received the highest amount of about £96.1 million [14].

This review aimed to investigate the prevalence and appropriateness of the statistical methods considered, in the planning and the analyses of cluster trials in practice for publicly funded trials, to evaluate the adherence by researchers to the reporting guidelines stipulated in the CONSORT 2010 statement for cluster trials and the recruitment abilities of cluster randomised controlled trials.

## Methods

### Search strategy

We manually searched through the online table-of-contents of each of the five NIHR journals, from 1 January 1997 to 15 July 2021 chronologically. The title and abstract of each report were screened to identify if a cluster randomised controlled trial was reported in it. If the title and abstract did not provide sufficient information to determine whether a cluster trial was reported, we had to read through the introduction and methodology chapters of the report to decide if the report should be included.

### Trial identification

To identify reports to be included in this review, we followed the procedure described in the “Search strategy” subsection. Apart from the HTA Journal that published its first volume in 1997, the other four journals are recent editions to the NIHR Journals Library. The HSDR, PGfAR and PHR journals published their first volume in 2013 while EME published its first volume in 2014. A search through the NIHR HTA archive from 1 January 1997 to 15 July 2021 showed that the first report of a cluster randomised controlled trial was published in 2000 [15]. However, choosing 1997 as the starting point enabled us to assess the adherence to the CONSORT reporting guidelines before and after the publication of the CONSORT 2010 statement extension for cluster trials. Our interest was solely on trials in which groups of individuals was the unit of randomisation.

One researcher (BCO) conducted the search and extraction of the information while two other independent reviewers (SJW and RMJ) supervised and validated a sample (25%) of the total trials reviewed. If the inclusion of a report was in doubt, this was discussed by all three reviewers until a consensus was reached. The cRCT reports were obtained from the NIHR Journals Library website (https://www.journalslibrary.nihr.ac.uk/#/ date last accessed 9 August 2021) along with any previously published trial paper, protocol paper or trial protocol, where available. For trials that had a published International Standardised Randomised Controlled Trial Number (ISRCTN) number, this was used to check the ISRCTN register of clinical trials for any additional information, a trial website or any previously unobtainable trial reports (cf. http://www.isrctn.com/). The trial reports published in the NIHR Journals Library were was used as the main resource where there were discrepancies in reporting. January 1997 was chosen as a start date for the review as this was the date of publication of the first report in the NIHR Journals Library (in the NIHR HTA Journal).

### Eligibility criteria

For a study to be eligible, it must be a cluster randomised controlled trial (involving the randomisation of groups of individuals) or stepped wedge cRCT published in any of the five online NIHR Journals library, from 1 January 1997 to 15 July 2021. Reports on all other study designs were excluded. Pilot and/or feasibility cRCTs were excluded as these have separate specific design and analysis issues including outcomes, sample size and statistical analysis and reporting. Full texts of identified reports were retrieved for further assessment.

### Patient and public involvement

Patients and/or the public were not involved in the design, or conduct, or reporting, or dissemination plans of our research.

### Data extraction

Once the NIHR Journals Library reports on cluster trials have been selected for inclusion, necessary information was extracted, using a standardised and piloted data extraction form. When the information of interest was not found, this was indicated with “Not Reported (NR)”; NR indicates that the author(s) did not consider or make use of the method/item of interest or might have used or considered the method/item of interest but did not report it.

The relevant information was extracted and stored in an Excel spreadsheet for further analysis. The information obtained was informed by the review of Walters et al .[16] and the relevant components for cRCTs as stipulated in the CONSORT 2004 statement and its subsequent update. These are the details of the article, information on sample size calculation, recruitment, follow-up, details on clustering, allocation, design/type of trial, primary outcome, primary analysis and results. An additional file presents the list and description (where necessary) of all the items extracted (see Additional file 1). The extracted information was analysed and reported in accordance with the Preferred Reporting Items for Systematic Reviews and Meta-Analyses (PRISMA) [17] guidelines where applicable (see Additional file 2 for the populated PRISMA checklist). In this review, the main outcome was the statistical methods used for analysing the primary outcome(s) of the cRCTs.

### Analysis

During the review, we identified that several of the individual reports in the NIHR Journals Library reported the results of two or more separate cRCTs [18–22], as well as the results for two or more primary outcomes per trial [21, 23–31].

Descriptive statistics using frequencies and percentages were generated for the levels of all the categorical characteristics of the trials reviewed, while mean, standard deviation, range, median and interquartile range were obtained for continuous outcomes. All analysis was done using an Excel spreadsheet (Microsoft® Excel for Mac, version 16.51) and R studio (Version 1.4.1717).

## Results

### Trial characteristics

**Fig. 1:**
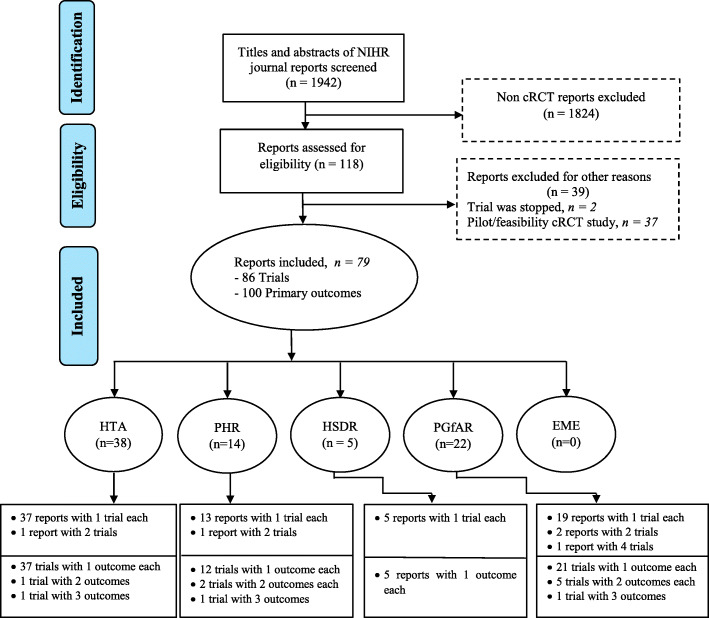
The search and selection process of cRCT reports from the five online NIHR Journals library surveyed from 1 January 1997 to 15 July 2021

Reports were extracted from the five online NIHR Journals Library published from 1 January 1997 to 15 July 2021. In total, 1942 reports were screened for eligibility, 118 cRCTs reports met the initial inclusion criteria and 3 of the reports were stepped wedge cRCTs [29, 32, 33]. Two reports were excluded because their trials were stopped due to poor recruitment, and only qualitative findings were thereby reported [34, 35. Thirty-seven other pilot/feasibility cRCTs were further excluded. Seventy-nine reports containing the results of 86 cluster trials were included. Five reports contained the results of multiple cluster trials (4 reports of 2 cRCTs each and 1 report included 4 cRCTs) 19–23. A total of 100 primary outcomes (11 trials in 10 reports had multiple primary outcomes) were assessed in this review. The search and selection processes are presented in Fig. 1. The list and URL of all included reports are available in a separate (Additional file 3).

Table 1 summarises the characteristics of the 86 trials included in this review. Most of the trials reviewed were conducted in different regions but solely within the United Kingdom (UK) except for Simmons et al. [35] which involved other European locations. The trials design used was mostly a parallel-group cluster trial that involved a direct comparison between intervention and control experimental arms (85%, 73/86), and this was mostly done using two experimental arms for comparison (80%) (Table 1).

**Table 1:** Characteristics of cluster randomised controlled trials published in the NIHR Journals Library, from 1 January 1997 to 15 July 2021

Characteristic	***n***	%
**NIHR journal the cRCT was reported in (*N*** **= 79**^**+**^**)**		
HTA	38	48
PHR	14	18
HSDR	5	6
PGAR	22	28
EME	0	0
**Trial design (*N*** **= 86)**		
Parallel	73	85
Factorial	7	8
Cross-over	2	2
Others*	4	5
**Number of trial arms (*N*** **= 86)**		
2	69	80
3	10	12
2 × 2	4	5
2 × 2 × 2	2	2
2 × 6	1	1
**Clinical area (*N*** **= 86)**		
Cancer/oncology	8	9
Mental health (including neurosciences/psychiatry/psychology/dementia)	21	25
Orthopaedics/rheumatology/musculoskeletal (including back pain)	2	2
Obstetrics and gynaecology	2	2
Primary care	6	7
Cardiovascular	1	1
Gastrointestinal	2	2
Respiratory	1	1
Stroke	4	5
Diabetes	6	7
Dermatology (including ulcers)	1	1
Others^†^	32	37
**Setting (*N*** **= 86)**		
Hospital	4	5
General practice	25	29
Mixed	3	3
Community	3	3
Others^‡^	51	59
**Level of clustering (*N*** **= 86)**		
2	85	99
3	1	1
**Trial registration (*N*** **= 86)**		
ISRCTN	78	91
NTC	2	2
Not reported	6	7
**Type of intervention (*N*** **= 86)**		
Therapy	8	9
Behaviour change technique	4	5
Complex intervention	17	20
Education	12	14
Exercise	3	3
Information and communication technology	3	3
Medical device	2	2
Screening	2	2
Training	17	20
Others^§^	18	21
**Type of control (*N*** **= 86)**		
Active	86	100
**Are patient blinded (*N*** **= 86)**		
Yes	8	9
No	78	91
**Any form of a pilot study**^**a**^ **(*N*** **= 86)**		
Yes	72	84
No	14	16
**Geographical region (*N*** **= 86)**		
Multiple regions	54	63
Regional	32	37

^a^These are internal pilot studies carried out within the main trials; they are different from the external pilot/feasibility studies mentioned initially in text

^+^79, the total number of journal reports included, which reported the results of 86 cRCTs (79 reports included the results of 86 cRCTs)

*Partial factorial and step-wedged trials

^†^Insomnia, paediatrics, youth bullying and other aggressive behaviours, traumatic brain injury, autism spectrum disorders, prehospital emergency care, obesity, epilepsy, oral health, end of life care, children fruit and vegetable intake, alcohol abuse, physical activity, psychosocial work environments, relationship and sexuality education, illicit drug use, smoking prevention, social and emotional wellbeing of children, dating and relationship violence, emergency admission to hospital, care for older people, multimorbidity, abdominal surgery, care of people with long-term conditions, care planning in secondary care mental health services and psychosis, eating disorder, injuries in under-fives children, patient involvement in safety, psychosis, care planning in secondary care mental health services

^‡^Care homes, nursing homes, clinics, NHS trust, residential services, stroke rehab unit, children centre, paediatrics diabetes clinic, schools, ambulances services, dental practice, stroke services

^§^Telephone triage, strategies to increase screening, financial incentive, invitation letter, leaflet, behavioural approaches, questionnaire, redesigned care model, health promotion, operational protocol, implementation package, time

### Statistical methods used for analysing cluster trials

Of the 100 primary outcomes reported in the 86 trials, the data type of most of the primary outcomes was continuous (63%, 63/100), followed by binary outcomes (28%), and then counts (5%), time-to-event [33, 36] and percentage [37, 38] were the least (2%, respectively). In the description of the statistical analysis of the primary outcomes of the cRCTs, a variety of phrases were used to describe the multilevel regression methods used to account for clustering, such as *generalized linear mixed-model*, *two-level hierarchical model*, *mixed-effects*, *multilevel regression* and *two-level heteroscedastic linear regression model*; hence, we used a generic name “generalized linear mixed model (GLMM)” to cover all the multilevel regression methods.

Of the 100 analysed primary outcomes in the trials, 80% (80/100) used a GLMM to account for clustering, 7% used regression methods with robust standard errors and 6% used generalized estimating equations (GEE) to estimate the regression coefficients of the models. Most of these analyses were carried out using individual participant outcomes. Only 2 trials used aggregated cluster level outcomes as data points in their primary analyses [31, 38]. The different statistical methods used to account for the clustering of outcomes at the analysis phase are presented in Table 2.

**Table 2:** Characteristics of the determinants of (and) the statistical methods used for analysing the primary outcomes in cluster trials

Characteristics	***n***	%
***Type of follow-up RCT (N = 86)***		
Closed cohort follow-up	76	88
Open cohort follow-up	4	5
Cross-sectional	4	5
Repeated cross-sectional	2	2
***Data type of primary outcome (N = 100)***		
Continuous	63	63
Binary	28	28
Counts	5	5
Time to event	2	2
Percentage	2	2
***Method of adjusting for clustering (N = 100)***		
Cluster-level analysis:		
Standard generalized linear model	2	2
Individual-level analysis:		
Generalized linear mixed model	80	80
Robust standard errors	7	7
Generalized estimating equations	6	6
Clustering not accounted for:		
Statistical hypothesis test statistic—chi-square	1	1
Standard generalized linear model	4	4
***Specific statistical model (N = 100)***		
Linear regression	57	57
Logistic regression	25	25
Analysis of covariance	6	6
Relative sensitivity	1	1
Negative binomial regression	2	2
Analysis of proportions	1	1
Cox Proportional Hazards model	2	2
Poisson regression	4	4
Weibull regression model	1	1
Chi-square test	1	1
***Random component of GLMM (N = 80)***		
Random intercept	76	95
Shared frailty	1	1
Random intercept and slope (repeated measures)	3	4
***Correlation structure in GEE (N = 6)***		
Exchangeable correlation	5	83
Correlation structure not reported	1	17

*N* = total number of trials; *n* = counts observed in each level of a category; RCT = randomised controlled trial; GLMM = generalized linear mixed model; GEE = generalized estimating equations. Not reported means that the information of interest was not considered and/or provided in the trial

Overall, 95% of the primary analyses used recognised statistical methods to adjust for clustering in their analyses, 5% did not; they ignored clustering and used standard statistical methods such as the *chi-square test*, *standard linear*, *logistic* and *Poisson regressions* [15, 29, 39–41]. Continuous outcomes were dichotomised in some studies to enable the use of logistic regression. The trial hypothesis was “superiority” in all the cluster trials except for Heller et al. [42] which was a non-inferiority trial. Table 2 also shows that most trials recruited and followed up the cohort of participants until the end of the trial; this often leads to missing data due to loss to follow-up (88%, 76/86).

Although 92% of the trials acknowledged the occurrence of missing data, most of them went ahead to analyse only complete cases (84%). Imputation of missing outcome data was done for just 16% of the trials reviewed [20, 28, 30, 43–53].

### Planned recruitment targets of participants and clusters

Recruitment characteristics are summarised in Table 3, with 67% (58/86) of cRCTs achieving their planned final individual participant recruitment target and 87% of the trials achieving ≥ 80% of their final individual participant recruitment target; this indicates successful recruitment to final targeted sample size for most of the cluster trials. This also applies to the original cluster recruitment target, with 89% of the trials successfully recruiting (and randomising) ≥ 80% of their original targeted number of clusters.

**Table 3:** Planned participants and clusters recruitment to targets in cluster trials

Characteristics	***n***	%	Mean (SD)	Median	Range	IQR
**Original individual participant target sample size (*N*** **= 84**^**b**^**)**						
≤ 300	11	13	10,035 (31357)	1250	136–250,000	550–4466
301–600	11	13				
601–900	13	15				
901–1200	3	4				
1201–1500	11	13				
1501–1800	3	4				
> 1800	29	35				
Not reported	3	3				
**Final individual participant target sample size (*N*** **= 84**^b^**)**						
≤ 300	11	13	9372 (30173)	1212	136–250,000	534–4258
301–600	11	13				
601–900	14	17				
901–1200	5	6				
1201–1500	11	13				
1501–1800	3	4				
> 1800	27	32				
Not reported	2	2				
**Original individual participant target sample size met (*N*** **= 86**)						
Yes	57	66				
No, but >= 80% met	14	16				
No and < 80% met	9	11				
**Final individual participant recruitment target met (*N*** **= 86)**						
Yes	58	67				
No, but 80% >= of target met	17	20				
No and < 80% of target met	6	7				
Not reported	5	6				
**Revised original individual participant target sample size (*N*** **= 86)**						
Yes, upward	13	15				
Yes, downward	9	10				
Yes, direction not reported	4	4				
No	61	71				
**Original cluster recruitment target met (*N*** **= 86)**						
Yes	68	79				
No, but >= 80% met	9	11				
No, and < 80% met	1	1				
Not reported	8	9				

^b^Two studies were excluded because the original and final targets were expressed in person-years of observation and not specific number of participants [41, 54]. *N* = total number of trials; *n* = counts observed in each level of a variable; SD = standard deviation; IQR = interquartile range. Not reported means that the information of interest was not considered and/or provided in the trial

### Cluster and sample size characteristics

In Table 4, the cluster and sample size characteristics of the included trials are summarised and presented. The design effect if not reported was calculated using the formula, 1 + (*m* − 1) × *ICC* or 1 + [(*CV*^2^ + 1)*m* − 1] × *ICC* for equal and unequal/varying cluster sizes respectively, where *CV* is the coefficient of variation, and *m* is the average cluster size. This is possible if the ICC and cluster size were reported. The median number of clusters randomised was 44 (IQR, 25–74), the minimum was 7 clusters randomised [42], and the maximum was 922 clusters randomised, in a trial of which households were the clusters [55]. A reasonable proportion of the randomised clusters were retained throughout the follow-up period, with a median of 43 clusters (IQR, 25–69) included in the analysis which is quite close to the number of clusters randomised. Also, for the number of subjects recruited/randomised, the median was 1184 (IQR, 597–3653), while the median number of subjects included in the analyses was 870 (IQR, 441–2356).

**Table 4:** Cluster and sample size characteristics of the trials included in the review

Characteristics	***n***	%	Mean (SD)	Median	Range	IQR
**No. of clusters randomised (*N*** **= 86)**						
4–10	2	2	77 (121)	44	7–922	25–74
11–20	11	13				
21–50	40	47				
51–100	21	24				
101–200	5	6				
> 200	7	8				
**No. of clusters analysed (*N*** **= 86)**						
0–10	2	2	76 (118)	43	7–864	25–69
11–20	12	14				
21–50	40	47				
51–100	21	24				
101–200	4	5				
> 200	7	8				
**No. of subjects recruited (*N*** **= 84**^**b**^**)**						
≤ 300	7	8	15,348 (48315)	1184	141–265,434	597–3653
301–600	14	17				
601–900	11	13				
901–1200	9	11				
1201–1500	9	11				
1501–1800	3	4				
> 1800	29	34				
Not reported	2	2				
**No. of subject analysed (*N*** **= 84**^**b**^**)**						
≤ 300	15	18	14,367 (48419)	870	42–264,325	441–2356
301–600	15	18				
601–900	13	15				
901–1200	5	6				
1201–1500	5	6				
1501–1800	2	2				
> 1800	25	30				
Not reported	4	5				
**Planned ICC for sample size (*N*** **= 86)**						
0.00–0.02	18	21	0.065 (0.082)	0.05	0.0002–0.5	0.0258–0.0700
> 0.02–0.05	33	38				
> 0.05–0.08	9	11				
> 0.08–0.11	8	9				
> 0.11–0.14	2	2				
> 0.14	6	7				
Not reported	10	12				
**Planned design effect (*N*** **= 86)**						
0.00–2.99	47	55	4.5 (8.90)	1.96	1.03–70.5	1.384–4.600
3.00–5.99	12	14				
6.00–8.99	10	12				
9.00–11.99	1	1				
≥ 12	3	3				
Not reported	13	15				
**Observed ICC of analysed primary outcome (*N*** **= 100)**						
− 0.02 to 0.02	35	35	0.06 (0.12)	0.02	-0.02 to 0.63	0.0010–0.0600
> 0.02–0.07	9	9				
> 0.07–0.12	3	3				
> 0.12–0.17	6	6				
> 0.17–0.22	2	2				
> 0.22	3	3				
Not reported	42	42				

^b^Two trials were excluded because the analysed subjects were measured in person-years. *N* = total number of trials and/or primary outcomes; *n* = counts observed in each level of a category; SD = standard deviation. Not reported means that the information of interest was not considered and/or provided in the trial

In the planning stage, 38% (33/86) of the planned ICCs used in the sample size calculations fell in the 0.03–0.05 range. The median planned ICC in the sample size calculation was 0.05 (IQR, 0.026–0.07). The observed ICCs from the analysed primary outcomes in the trials has a median value of approximately 0.02 (IQR, 0.001–0.060) with most of the reported ICCs occurring in the − 0.02 to 0.02 range (Table 4). After excluding two trials that were analysed at the cluster-level, we found that 42% (42/100) of the observed ICC from the primary analyses of the primary outcomes were not reported. Thirty-one percent of the observed ICC was not reported before the publication of CONSORT 2010 statement compared to 44% after its publication (Table 4). One study carried out a pair matched randomisation using a minimisation technique; however, they analysed their primary outcomes at the individual-level [28]. Pair matching of clusters reduces the population heterogeneity at the cluster level which could result in a negligible ICC from the analysed primary outcome and also improve the statistical efficiency of the trial [8, 10].

Not reporting the observed ICC for the analysed primary outcomes contradicts the CONSORT 2010 reporting guidelines for cluster trials, which recommends that authors should report “a coefficient of intracluster correlation (ICC or k) for each primary outcome”. The minimum observed ICC value appears to be an outlier (− 0.02) and was found in Heller et al. [42].

**Fig. 2:**
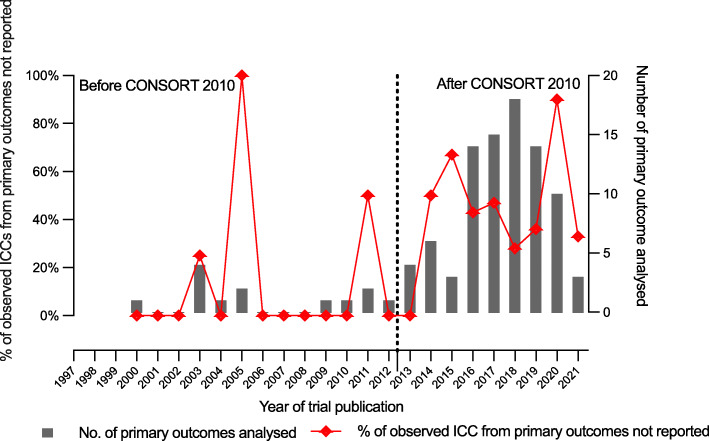
Plot comparing the trend of not reporting the observed ICCs of analysed primary outcomes in cRCTs before and after CONSORT 2010 statement with the first published cRCT in NIHR Journals library recorded in 2000

Figure 2 shows the trend and comparison of the practice of not reporting the observed ICCs for the analysed primary outcomes, before and after CONSORT 2010 statement. No observable trend appears to be present in Fig. 2. Before the publication of CONSORT 2010 guidelines for cRCT, the years that trials were carried out, 2003, 2005 and 2011 also recorded non-reporting of the observed ICCs for the analysed primary outcomes (20%, 100% and 50%, respectively). However, after the publication of the CONSORT 2010 statement, almost in each year aside 2013, some of the observed ICCs for the analysed primary outcomes were not reported, ranging from 28 to 90%. From Table 5, a higher proportion still did not report their observed ICCs from analysed primary outcomes after the publication of the CONSORT 2010 statement compared to the proportion that did not before its publication (44% vs 31%).

**Table 5:** Comparison of the non-adherence in the reporting of observed ICC for each primary outcome before and after CONSORT 2010 statement for cRCTs (published in Sept 2012)

	Year of publication		
	Before 1997–2012	After 2013–2021	All 1997–2021
Number of trials	11	75	86
Number of primary outcomes	13	87	100
Number of primary outcomes with the observed ICC not reported (%)	4 (31)	38 (44)	42(42)

## Discussion

This review was carried out to investigate the statistical methods used for analysing cluster randomised controlled trials in practice; to this end, we surveyed publicly funded cluster trials funded by the National Institute for Health Research.

Most of the trials used appropriate/recognised statistical methods to adjust for clustering in the main analyses of the primary outcomes from the trials (95%, 95/100). Few of the outcomes (and trials) 5% ignored clustering and used standard statistical methods that assumed independence among outcomes from participants in a cluster. This approach is not reccommended as it could lead to smaller standard errors and consequently, an increased value of the test statistic, smaller *P*-values, narrower confidence interval and possibly increase the type I error rate compared with the statistical methods that allow for clustering. If this happens to be the case, misleading conclusions and decisions will be made; this could have detrimental effects on public health.

The generalized linear mixed model (GLMM) was the most popular choice in adjusting for clustering and was more popular than GEE (80% vs 6%). For the GLMMs with two levels of clustering (trial participants nested within clusters), the cluster unit is usually incorporated as a random intercept to account for clustering. Where the primary outcome was measured more than once or the level of clustering is more than two levels, statistical models with random intercept and the random slope were used. Four trials that used the GEE method assumed an exchangeable working correlation structure in the primary analysis [18, 56–58], while 1 trial did not report the correlation structure that was assumed [42].

Fiero et al. [59] conducted a systematic review that focused more on the handling of missing data than on the statistical methods used for analysing cluster trials and found similar results to ours. They found that most of the trials analysed their primary outcome using GLMMs, and the cluster unit were modelled as the random intercept to account for clustering. Also, they found that all 14 (100%) of the trials that used GEE to account for clustering assumed an exchangeable correlation structure, which is similar to the findings of this current review (5/5, 100%; one study did not report their correlation structure [42]). Overall, they found that a lower proportion 79% (68/88) of the trials accounted for clustering compared to our review which observed a higher proportion 95% (95/100).

It is worth noting that while the use of appropriate statistical methods is high, none of the trials considered the recent potentially improved statistical methods developed in other study designs where clustered data do arise, such as the quadratic inference function (QIF), the alternating logistic regression (ALR) and the targeted maximum likelihood (tMLE). These recent methods are improvements over the standard GEE method for estimating the regression coefficients in the model [60]. The results of our study revealed that the number of clusters randomised in a cRCT could be as large as 922 in a study where the clusters were households [55] and as few as 7 clusters [42]. This result is different from the findings of Arnup et al. [61] where they focused on cluster randomised cross-over trials, one reason for choosing a cross-over design is if the number of the prospective clusters is small. In their study, the lowest number of clusters randomised was 2 while 25% of the number of clusters randomised was below 4.

In practice, active controls are mostly used when assessing the effect of non-pharmacological interventions (86/86, 100%). As revealed in our results, most times, it is impractical to conduct studies where the participants are blinded to the experimental arms they are allocated to. However, to some extent, masking is achieved by blinding either the person randomising the subjects, the assessor and/or the statistician that will analyse the data. To carry out a robust cluster trial, it is preferable to conduct an internal pilot/feasibility study (84%, 72/86) to assess the viability of the items/phases of the trial, such as the data collection tools, the understanding (and safety) and acceptance of the intervention by the participants and the ability to recruit to target before proceeding with the main trial.

Recruiting participants (for clusters, see Table 3) into a trial seems not to be a problem in cluster trials, particularly when compared to iRCTs (see Table 6). In 87% of the cluster trials, researchers were able to recruit ≥ 80% of their final planned participant recruitment targets. This result also applies to the number of clusters recruited/randomised, where 76% of the trials were able to recruit ≥ 80% of their planned clusters recruitment target (see Table 6).

**Table 6:** Comparing the ability to recruit to the target the number of participants between cRCTs and iRCTs using results of previous studies that reviewed iRCTs

Review	McDonald et al.	Sully et al.	Walters et al.	This study
Recruitment period	1994–2002	2002–2008	2004–2016	1997–2021
Number of trials in the study	***N*** = 122 iRCTs	***N*** = 73 iRCTs	***N*** = 151 iRCTs	***N*** = 86 cRCTs
Recruited 100% of original target	38 of 122 (31%)	40 of 73 (55%)	61 of 151 (40%)	**57 of 86 (66%)**
Original target was revised	42 of 122 (34%)	14 of 73 (19%)	52 of 151 (34%)	21^c^ of 86 (24%)
Original target revised upward	6 of 42 (14%)	5 of 14 (36%)	11 of 52 (21%)	**12 of 21 (57%)**
Original target revised downward	36 of 42 (86%)	9 of 14 (64%)	41 of 52 (79%)	9 of 21 (43%)
Recruited 80% of original target	67 of 122 (55%)	57 of 73 (78%)	95 of 151 (63%)	**71 of 86 (83%)**
Recruited 100% of revised target	19 of 42 (45%)	10 of 14 (71%)	28 of 52 (54%)	**16 of 21 (76%)**
Recruited 80% of revised target	34 of 42 (80%)	13 of 14 (93%)	48 of 52 (92%)	**21 of 21 (100%)**
Extended their recruitment	65 of 122 (54%)	33 of 73 (45%)	49 of 151(32%)	**11 of 86 (13%)**

Source: Adapted (and modified) from Walters et al. [16]

^c^Was supposed to be 25 trials but 2 trials did not report their original target that was revised, and another two trials did not report their final revised target and the number of participants recruited respectively; they were excluded since comparison cannot be done

In Table 6, we compared the ability of cRCTs and iRCTs to recruit to their target the number of participants. In terms of recruiting to 100% of the original participant target, cluster trials seem more successful than iRCTs (66% vs 55%). This is confirmed by the fact that in cluster trials, the originally planned sample sizes are rarely revised (24%) and tend to be revised upward (57%, 12/21) rather than downward (43%). When compared to iRCTs, the number (and percentage) of upward revisions were higher in cluster trials (57% vs 36%). Even with the most upward revisions, cluster trial recruitment periods are rarely extended to meet up with recruitment targets compared to iRCTs (13%, 11/86 vs 54%, 65/122).

We also found that in practice the completely randomised parallel-group cluster trial design is the most used design involving two experimental arms in its simplest form. This cluster design is easy to set up, implement and analyse. Our results indicated that all the trials reviewed, except one, were superiority trials involving contrasting experimental arms. For the sample size calculation, our results indicated that the median assumed ICC value, used in the calculation, was 0.05, while the average was 0.065. However, we found observed that the ICC assumed in the sample size calculation could be as low as 0.0002 (Table 4). Our results also indicated that a disappointing trend of not reporting the observed ICC for each primary outcome is happening. About 4 out of 10 of the observed ICCs from the analysed primary outcomes in cRCTs are not being reported. The implication of not reporting the ICC cannot be overemphasised; the ICC is an important item in designing/planning future cluster trials as it is needed for sample size calculation. It is reasonable to make it available for researchers planning to undertake similar study. The importance of reporting the ICC was reemphasised by the development of a framework specifying how and what should be reported in association with the ICC to facilitate understanding and the planning of future cluster trials [62].

Surprisingly, this occurs more in recent times despite the availability and publicity of the CONSORT 2010 statement extension for cluster trials, although 2005 had the highest percentage of this disappointing practice (100%), but with only two analysed primary outcomes recorded.

It is worth noting that this was also after the publication of the CONSORT 2004 statement [8] extension for cluster trials. Ivers et al. [11] assessed the impact of the CONSORT 2004 statement extension for cluster trials on quality of reporting and study methodology, and one of the criteria compared was the “reporting of an estimated intracluster correlation”. They found only 18% of the 300 manuscripts reported an ICC estimate and 22% vs 14% before and after CONSORT 2004 statement respectively. This result indicated a decline in the practice of reporting indicated a decline in the practice of reporting the observed ICC which is similar to our current study. We found a 13% increase (change in non-adherence before to after CONSORT 2010) in non-adherence to the CONSORT reporting guidelines with regards to reporting the observed ICC for each primary outcome analysed, using CONSORT 2010 statement as the basis for comparison. CONSORT statements extensions for cluster trials are published to facilitate improved quality reporting of cluster trials. If used properly, they are supposed to help in the understanding, assessing and replicating of cluster trials by all stakeholders of clinical trials. Hence, all authors intending to write up the report for their cluster trial(s) should make good use of the updated CONSORT 2010 statement.

The observations made in this review is that in practice there are important issues in cRCTs that are still being ignored or handled inadequately or not reported.

Firstly, missing data is not adequately handled most of the time. The majority (79/86, 92%) of the studies reviewed acknowledged the existence of missing data, which is obvious due to inevitable loss to follow-up in a closed cohort follow-up study; however, the majority still went ahead to analyse only available observations (84%). To assess the robustness of the findings in the trials, especially when missing data was not technically handled, most researchers resorted to conducting sensitivity analysis. However, if they had handled the problem of missing data technically (e.g., using statistical imputations) in the original analysis, it could have improved the inferences made in the study.

Secondly, there appears to be a slow uptake of improved statistical methods developed in other study designs where clustered data can arise, such as the QIF, tMLE and ALR that are potentially better in certain situations than the popular statistical methods used currently for analysing cluster trials. It would be ideal if these methods are publicised by methodologists of cluster trials so that researchers can use them when necessary to make optimal inferences [60].

## Limitation

We acknowledge that searching and retrieving cluster trial reports from one source could lead to publication bias. We optimized the study by including all cluster trials instead of a random sample, and the reports published in the NIHR Journals Library were also published independently as result articles in other journals; hence, reports included in this review represent a collection of articles from several journals independent of NIHR Journals Library.

## Conclusion and recommendation

In practice, most of the publicly funded cluster trials adjusted for clustering using an appropriate/recognised statistical method with most of the primary analyses done at the individual level using generalized linear mixed models. However, the inadequate analysis and poor reporting of cluster trials, particularly not reporting the observed ICC for the analysed primary outcomes is still happening in recent times despite the availability of the CONSORT reporting guidelines extension for cluster trials published over a decade ago. One way of addressing this issue is to encourage journal editors and peer reviewers to insist that authors should adhere to CONSORT reporting guidelines for cluster trials when submitting their manuscripts. This review will serve as a reference tool in conducting systematic reviews of statistical methods used in practice and statistical methods available in the literature for analysing cluster trials.

### Patient and public involvement

Patients and/or the public were not involved in the design, conduct, reporting or dissemination plans of this research.

## Supplementary Information

**Additional file 1.** Data collection tool (NIHR).

**Additional file 2.** PRISMA 2020 checklist.

**Additional file 3.** List of included reports.

## Data Availability

The information extracted in this review is based on published trials in the NIHR Journals library. The data extracted from the NIHR Journals Library supporting the finding of this study is available upon reasonable request from the corresponding author.

## Abbreviations

cRCT: Cluster randomised controlled trial
CONSORT: Consolidated standards of reporting trials
NIHR: National institute for health research
SD: Standard deviation
ICC: Intracluster correlation coefficient
IQR: Interquartile range
RCT: Randomised controlled trial
iRCT: Individually randomised controlled trial
GEE: Generalized estimating equation
GLMM: Generalized linear mixed model
QIF: Quadratic inference function
tMLE: Targeted maximum likelihood
ALR: Alternating logistic regression
PHR: Public health research
HSDR: Health services and delivery research
EME: Efficacy and mechanism evaluation
PGfAR: Programme grants for applied research
HTA: Health technology assessment
NR: Not reported
